# Does the Knowledge of the Local Thickness of Human Ascending Thoracic Aneurysm Walls Improve Their Mechanical Analysis?

**DOI:** 10.3389/fbioe.2019.00169

**Published:** 2019-07-16

**Authors:** Cristina Cavinato, Jerome Molimard, Nicolas Curt, Salvatore Campisi, Laurent Orgéas, Pierre Badel

**Affiliations:** ^1^Mines Saint-Etienne, Centre CIS, INSERM, U 1059 Sainbiose, Univ Lyon, Univ Jean Monnet, Saint-Etienne, France; ^2^Department of CardioVascular Surgery, CHU Hôpital Nord Saint-Etienne, Saint-Etienne, France; ^3^UMR 5521, Univ. Grenoble Alpes, CNRS, Grenoble INP, 3SR Lab, Grenoble, France

**Keywords:** ascending thoracic aortic aneurysm, aortic wall, local thickness, material heterogeneity, sDIC

## Abstract

Ascending thoracic aortic aneurysm (ATAA) ruptures are life threatening phenomena which occur in local weaker regions of the diseased aortic wall. As ATAAs are evolving pathologies, their growth represents a significant local remodeling and degradation of the microstructural architecture and thus their mechanical properties. To address the need for deeper study of ATAAs and their failure, it is required to analyze the mechanical behavior at the sub-millimeter scale by making use of accurate geometrical and kinematical measurements during their deformation. For this purpose, we propose a novel methodology that combined an accurate tool for thickness distribution measurement of the arterial wall, digital image correlation to assess local strain fields and bulge inflation to characterize the physiological and failure response of flat unruptured human ATAA specimens. The analysis of the heterogeneity of the local thickness and local physiological stress and strain was carried out for each investigated subject. At the subject level, our results state the presence of a non-consistent relationship between the local wall thickness and the local physiological strain field and high heterogeneity of the variables. At the inter-subject level, thicknesses were studied in relation to physiological strain and stress and load at rupture. The rupture pressure was correlated with neither the average thickness nor the lowest thickness of the specimens. Our results confirm that intrinsic material strength (hence structure) differs a lot from a subject to another and even within the same subject.

## Introduction

Aneurysm rupture involving thoracic aorta is one of the principal causes of vascular disease-related death, accounting for an annual age- and sex-adjusted incidences of 3.5 per 100,000 individuals. Anatomically, these aneurysms are ascending thoracic aortic aneurysm (ATAA) in 85% of the cases (Clouse et al., 2004). Mortality rate from thoracic aortic rupture is extremely high, i.e., 97 to 100% (Johansson et al., 1995) and surgical repair by the resection with a prosthetic graft is the current therapy for this pathology. At present, there is no critical trigger factor that permits to predict impending rupture. Therefore, most operative interventions are usually done when ATAA orthogonal diameter surpasses 55 mm (Svensson et al., 2013), when the rate of growth increases more than 0.5 cm/year (Boodhwani et al., 2014) and/or when serious aneurysm-associated symptoms occur (Lobato and Puech-Leão, 1998; Ramanath et al., 2009).

ATAA wall is exposed to the complex patient-specific hemodynamics created in the left ventricle outflow tract and it exhibits a complex solid mechanical behavior which is still poorly understood. Aortic mechanical strength stems from the content and architecture of cellular and matrix constituents, i.e., vascular smooth muscle cells (SMC), elastin, collagen, and ground substance, which vary greatly within the thickness of the wall (Weisbecker et al., 2013; Schriefl et al., 2015) and locally in all directions (Cavinato et al., 2017).

Rupture of ATAAs can be considered as the effect of excessive mechanical stresses or strains on a localized point of a diseased wall which is affected by abnormal microstructural remodeling of the vessel (Humphrey et al., 2014): disorganized distribution of collagen fibers (Tang et al., 2005; Cavinato et al., 2017) and fragmented elastin network (Phillippi et al., 2014; Pasta et al., 2016) with a decreased amount of elastin (Iliopoulos et al., 2009b; Lindeman et al., 2010) and SMC loss were observed in ATAAs if compared to the non-aneurysmal wall. For instance, the spatial variation of collagen and elastin fibers has a direct consequence on the evolution of the vessel wall stress and strain localization, which are relevant aspects in understanding the degeneration process and assessing aneurysm rupture risk (Vorp, 2007). In light of this background, patient-specific mechanical analysis should be able to provide accurate wall elastic properties distributions at the spatial scale of the local heterogeneities of the wall before making assumption in finding rupture risk indicators. Researchers started to focus on the local heterogeneities of the *ex vivo* biaxial behavior in ATAA specimens (Choudhury et al., 2009; Shahmansouri et al., 2016). However, as commonly achieved, the specimen thickness was an average estimation of multiple *ex vivo* measurements which can induce important discrepancies in the estimated stress fields. Thickness heterogeneity is a critical issue according to the most recent articles (Davis et al., 2016; Farotto et al., 2018). In particular, it has a central role in properly assessing the local stress field in the ATAA wall.

Within this context, the goal of this study is to propose an original experimental methodology, which combines for the first time both a highly accurate method to map the local initial thickness of the tissue and a method to reconstruct the distribution, with a sub-millimetric spatial resolution, of the mechanical stress-strain state of arterial tissues which were pressurized *ex vivo* using bulge tests. The method was applied to analyze the heterogeneous local thickness and physiological mechanical characteristics of human ATAA specimens, and the relationships between thickness and the physiological and rupture mechanical data and the clinical data.

## Materials and Methods

### Specimen Preparation

**Figure 1:**
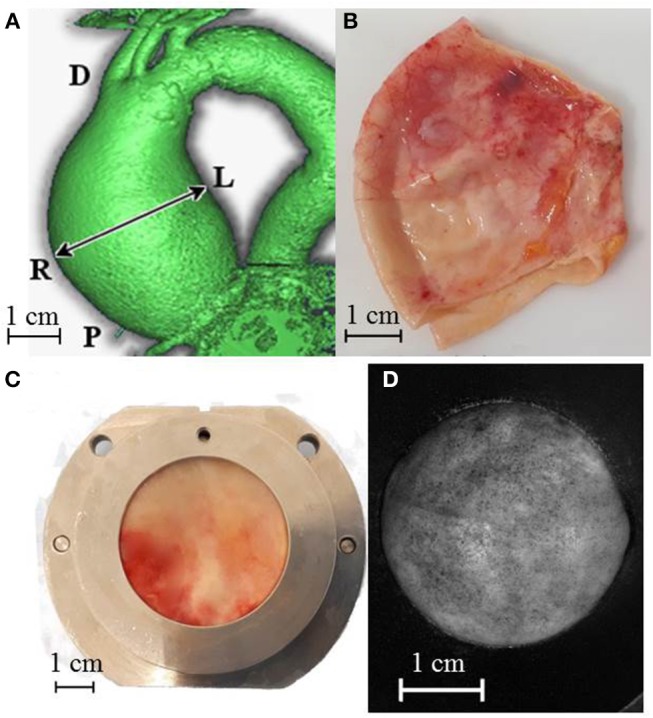
**(A)** CT dynamic scanner image indicating *in vivo* lumen diameter measure and adopted position references *D* = distal, *P* = proximal, *L* = left, *R* = right. ATAA specimen preparation and test steps: entire cut ATAA **(B)**, specimen positioned into the iron holder for the thickness measurement **(C)** specimen covered with graphite dots and inflated at a pressure of 250 mmHg. Its deformed shape was captured by one high-resolution camera during the mechanical test **(D)**.

Unruptured ATAA tissues (patients = 19, specimens = 20) were collected from patients undergoing surgical replacement in accordance with the French regulations and approved by the ministry of research, with written informed consent from all subjects. All subjects gave written informed consent in accordance with the Declaration of Helsinki. The protocol was approved by the ethics committee France Sud-Est 1. The study population included specimens of patients of both genders with ordinary ranges of age and *in vivo* ATAA diameter, as reported in Table 1, and no history of genetic connective disorders. Previously measured values for the *in vivo* lumen diameter of each ATAA at its point of maximum dilatation in diastolic phase were derived from CT dynamic scanner examination performed the day before the surgery by a trained expert (Figure 1A). A disc-shaped wall specimen with a diameter of about 45 mm was cut from the central part of the outer curvature of each ATAA (Figure 1B). One ATAA segment with a particularly large size could be separated in two specimens. The same specimen orientation with respect to the experimental set up was employed for all tests presented hereafter. After excision, the specimens were stored in PBS at 4°C and tested within 24 h of the operation.

**Table 1:** Shareable clinical information for collected ATAA specimens.

**Specimen**		**Age range (years)**	***In vivo* diameter (mm)**	***Ex vivo*** **thickness**			
				**Mean (mm)**		**sd (mm)**	
1		60–70	50.0	3.20		0.71	
2		50–60	59.0	3.63		1.36	
3		60–70	44.3	2.87		0.45	
4		70–80	57.7	3.56		0.62	
5		50–60	57.3	3.40		0.77	
6		60–70	59.0	3.76		0.96	
7		30–40	54.0	3.50		0.94	
8		70–80	54.3	2.84		0.40	
9		70–80	56.3	3.01		0.70	
10		60–70	46.0	3.52		0.96	
11		50–60	54.7	3.94		1.23	
12		70–80	49.7	2.33		0.37	
13		70–80	54.6	3.11		0.85	
14		70–80	54.0	2.55		0.44	
15		80–90	52.0	2.43		0.63	
16		70–80	55.0	2.76		0.55	
17		70–80	50.0	2.75		2.00	
18		80–90	54.0	2.36		0.32	
19	a	80–90	67.3	3.39	3.41	0.57	0.59
	b				3.37		0.56

Specimens were clamped between two 30-mm-inner-diameter PVC supports being held together until the end of all measurements and tests. These supports were mounted first onto the thickness measurement set-up and sequentially onto the bulge inflation set-up (Figures 1C,D, respectively) while ensuring the inalterability of the specimen region of interest and orientation. Further details of the specimen preparation were reported in an earlier publication (Cavinato et al., 2017).

Thickness measurements were performed for all the 20 specimens, while only 12 out of 20 could successfully be analyzed using the mechanical inflation test.

### Thickness Measurement Protocol

**Figure 2:**
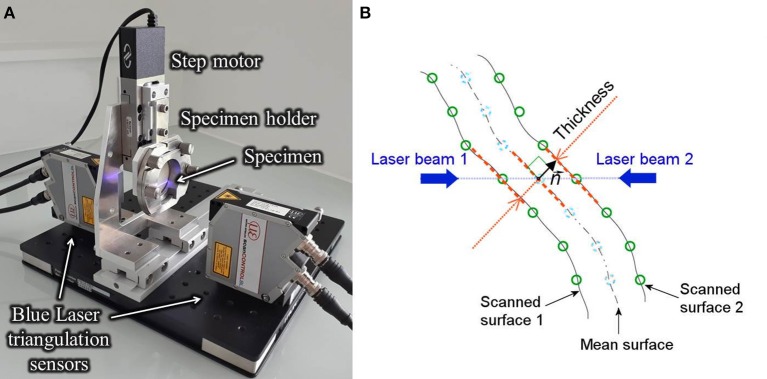
View of the thickness measurement test bench into action showing the line laser triangulation sensors and the central body consisting of motor and iron holder **(A)** and view of the cross section of the specimen indicating the principal elements of the thickness calculation procedure **(B)**.

The thickness measurement set-up, presented in Figure 2A, was made up of two high spatial resolution line laser triangulation sensors (optoNCDT 1700BL, Micro-Epsilon Messtechnik GmbH & Co. KG, Germany), headed toward each other in a horizontal line, and a central body midway between the sensors. The central body included a metallic holder and a linear translation stage (MFA-PPD, NewPort, CA, USA) designed to move the specimen along the vertical direction. Blue light (λ = 405 nm) lasers were chosen because soft tissues reflect this wavelength without significant light absorption and blur, as demonstrated for skin (Boyer et al., 2013). The scanner exposure time was 1 ms and the measurement field was 1280 × 768 pixels. A program developed in LabVIEW (National Instruments, Austin, USA) allowed recording the position profiles of both exposed surfaces of the specimen with a vertical step of 50 μm and a lateral resolution of 20 μm. To adjust the alignment error between the two laser sensors, a calibration procedure was carried out using 4 gage blocks, with thicknesses from 1 to 2.5 mm. Results showed a bias error on the thickness of 9 μm and a standard deviation of 12 μm. Measurements on each ATAA specimen were repeated once (total acquisition time of about 3 min). Thickness was estimated with a Matlab® program, by (i) mapping one surface onto the other, (ii) identifying the midpoints between points with the same planar coordinates on the two surfaces as mean surface, (iii) defining the vector normal $\vec{n}$ to the mean surface and (iv) calculating the thickness as the normal projection of the out-of-plane distance between the two scanned surfaces as schematized in Figure 2B.

### Mechanical Testing Protocol

Once the thickness measurement was performed, the specimens were mounted onto the bulge inflation device with the orientation being indexed on the device orientation as fully described in our previous paper (Cavinato et al., 2017). The circular exposed surface was sprayed with an aerosol of graphite that created a random speckle of black dots with a diameter of 0.10 ± 0.04 μm (see Figure 1C). The setup was then positioned in front of two high-resolution cameras (GOM, 5M LT) set for surface Stereo Digital Image Correlation (s-DIC). Part of the following protocol was drawn up and detailed by Romo et al. (2014). A volume controlled water pump pressurized the hermetic cavity formed between the specimen and the inflation system at a constant rate of 10 mL/min, until the tissue ruptured. The cameras and a pressure transducer were triggered together to get a measure every 1 s. Prior to the inflation which led to rupture, each specimen was preconditioned through five loading and unloading cycle up to 50 mmHg at the same inflation rate. The s-DIC was carried out using Aramis® software to measure the 3D displacement field of the speckled surface. To ensure a good performance between spatial resolution and matching noise, 21-pixel-facets, and 5-pixel-overlaps were chosen for capturing the actual deformation field of about 15,000 points on the circular surface (Reu, 2012). The results rely on precedent studies of the presented test bench showing a strain noise floor level of 0.6% (Davis et al., 2015).

Each protocol presented in this paper was performed by the same operator and in a sequential and continuous way to prevent specimens from dryness effects.

### Mechanical Data Reconstruction

A 3-D triangulated mesh was created by morphing a 3-D Delaunay template onto the undeformed 3D coordinates of the specimen's surface. A mesh of 1,203 elements and 644 nodes was chosen for the calculation and a natural neighbor interpolation was used to compute the displacement values of the mesh nodes and to assign the initial thickness value of the elements. The resulting node clouds, obtained at each loading increment, were used to compute the deformation gradient **F** on the upper surface of the tissue, followed by the Green-Lagrange strain tensor **E**. Then a method based on membrane elasto-statics was used to compute the stress field on the whole mesh as described earlier (Romo et al., 2014). Briefly, by considering an arbitrary dividing surface determined by vector position $\overset{}{x}$(ξ^1^, ξ^2^) where ξ^α^ are the curvilinear coordinates (α = 1, 2 are the 2 directions tangential to the surface) in the current configuration at each point of the surface, a pair of independent covariant tangential basis vectors were defined as ${\vec{g}}_{\alpha }=\frac{\partial \overset{}{x}}{\partial \xi^{\alpha }}$. The contravariant tangential basis vectors ${\vec{g}}^{\alpha }$ were then defined with the following conditions: ${\vec{g}}_{\alpha }{\cdot \;\vec{g}}^{\beta }=\delta_{\alpha }^{\beta }\;\left(\beta \;=1,2\right)$, $\delta_{\alpha }^{\beta }$ being the components of symmetric unit tensor. If ${\vec{G}}^{\alpha }$ and ${\vec{G}}_{\beta }$ are the covariant and contravariant metric vectors in the undeformed configuration, the deformation gradient tensor on the surface of the specimen can be defined as $\text{F}=\;{\vec{g}}_{\alpha }⊗\;{\vec{G}}^{{}^{\alpha }}$. Consequently, the Green-Lagrangian strain tensor was computed as follows:

(1) $$E=\frac{1}{2}\left(F^{T}F-I\right)=\;\frac{1}{2}\left({\vec{g}}_{\alpha }\cdot {\vec{g}}_{\beta }-{\vec{G}}_{\alpha }\cdot {\vec{G}}_{\beta }\right){\vec{G}}^{\alpha }⊗{\vec{G}}^{\beta }$$

Under the membrane assumption, the Cauchy membrane stress tensor **σ** was then computed from the static equilibrium problem (Lu et al., 2008) according to the following equation:

(2) $$\begin{array}{r} \frac{1}{\sqrt{g}}\;\frac{\partial \left({\sqrt{g}\;h\;\sigma }^{\beta \alpha }{\vec{g}}_{\alpha }\;\right)}{\partial \xi^{\beta }}+p\vec{n}=\vec{0} \end{array}$$

where *g* = det$\left({\vec{g}}_{\alpha }\cdot {\vec{g}}_{\beta }\right)$, *p* is the applied bulge pressure, $\vec{n}$ is the unit vector normal to the surface in the current configuration. Equation (2) was solved for the centroids of the mesh elements. Then the system was completed with boundary condition equations which (i) balanced the resulting force due to the pressure applied onto the inner surface and (ii) permitted no shear along the boundaries. A more complete description of the determination of the stress field and a finite element validation study is given in Romo (2014).

The actual thickness *h* of each element was also calculated for each deformed configuration using the initial thickness field *h*_0_ (measured) and the incompressibility assumption:

(3) $$\begin{array}{r} h=\frac{h_{0}}{F_{11}F_{22}-F_{21}F_{12}} \end{array}$$

where *F*_*ij*_ are the components of ***F*** in $\left({\vec{g}}^{1}{\vec{g}}^{2}\right)$.

For comparative purposes, Equation (2) was solved a second time using a constant thickness for all mesh nodes, denoted as ${\overset{\bar{}}{h}}_{0}$, average of the specimen initial thickness field. In this way, the method proposed by Romo et al. and based on a constant thickness value was reproduced by using an optical technique instead of an average of caliper measurements.

From this stress reconstruction method, the fields of the maximum principal strain E^*^ and the maximum principal stress σ^*^ were analyzed more precisely in the following. Their values were extracted at different relevant points, namely the points of their maximum and minimum values, the points of maximum and minimum thicknesses and the point at the top of the inflated specimen. Their average values were also considered.

### *In vivo* Pressure Calculation

In order to compare the mechanical fields of different specimens at a physiologically-meaningful specific load, the pressure measured in the inflation test was converted to an equivalent *in vivo* pressure, denoted as p_*vivo*_. In fact, it is important to point out that the blood pressure should not be considered equal to the applied pressure in the inflation test due to the change of shape between the ATAA *in vivo* and the inflation system. Therefore, the proposed methodology consisted in (i) fitting a sphere with a diameter d_*fit*_ to the deformed coordinates of the inflated specimens and (ii) using Laplace's law to find the equivalent pressure *in vivo*. The sphere fitting was performed with an iterative Gauss-Newton algorithm. Under the membrane theory assumption previously verified (Romo et al., 2014) and modeling the ATAA wall as a hemispherical membrane, the *in vivo* pressure for each inflation step can be written as:

(4) $$\begin{array}{r} p_{vivo}=\;\frac{p\;d_{fit}}{d_{vivo}} \end{array}$$

Where *p* is the applied pressure and *d*_*vivo*_ is the *in vivo* lumen diameter of the specimens from pre-surgical CT scans (Table 1). The purpose of this approach is to take into account the *in vivo* conditions of the tissues to permit the analysis of the stress and strain fields at a comparable load among specimens which have different *in vivo* diameters.

### Statistical Analysis

The repeatability in the thickness measurements was assessed by performing a non-parametric paired Wilcoxon signed-rank test (Lowry, 1999) of the thickness dataset and its repetition.

Following this, the first thickness dataset was used for the analysis and basic statistic values of the thickness maps were calculated. Since there were multiple specimens supplied from one of the patients, both patient and specimen statistics were determined. Using all datasets, the intra-patient variability was defined as the square root of the average of the patient-specific variances. The inter-patient variability was calculated as the standard deviation of the patient-specific average thickness values.

The linear correlation between local thickness and rupture pressure was analyzed with a non-parametric Spearman rank order test (Student, 1921) (the correlation coefficient is referred to as ρ).

The choice of non-parametric tests is due the hypothesis of normality of the data which was rejected when applying the Shapiro-Wilk normality test (Shapiro and Wilk, 1965). For assuming the linear regression, the normality assumption of the residuals, their mean equal to zero, and their constant standard deviation were verified.

The considered statistical test was performed using a threshold of 0.05 on the significance indicated as *p-value*.

## Results

### Thickness Characterization

**Figure 3:**
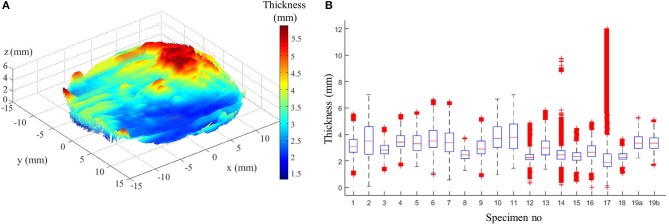
**(A)** Example of thickness measurement from patient no 4. **(B)** Box plot of all specimen thicknesses showing complementary statistics: median (red line), 25th and 75th percentiles (blue lines), most extreme data points not considered outliers (black lines), and outliers (red crosses).

Figure 3A shows a representative example of thickness measurements obtained from ATAA specimens. The spatial variability is highlighted by the color scale which ranged between 0.1 and 5.2 mm in this specific specimen. In a first instance, the repeatability analysis estimated by a Wilcoxon signed-rank test for paired variables showed no difference (*p-value* < 0.05) between the first and second measurement of all the specimens. The thickness measurements were thus considered as repeatable. In Table 1, the averages (over all measurement points) of the thickness measurements for each specimen are shown. For all patients, the mean thickness was 3.10 mm and there were an intra-patient variability of 0.88 mm and an inter-patient variability of 0.50 mm.

The case of two specimens sourced from the same patient no 19 revealed an inter-specimen variability of 0.03 mm. The two specimens of patient no 19 represent the left (a) and the right (b) side of the outer curvature of the ATAA where the left/right labels indicate the anatomical position (Figure 1A). The left specimen was slightly thicker than the right one (+1%, which is almost the range of error of the device). Complementary statistical information given in Figure 3B, i.e., medians, principal percentiles, and outliers, illustrate the intra-specimen variability in all specimens. There is evidence of a large intra-specimen variability, which was particularly emphasized in some specimens where a greater fibrotic and calcified appearance of the surfaces manifested by a higher number of outliers (e.g., in specimens no 14 and 17).

### Mechanical Analysis at Physiological Load

The bulge inflation test was considered successful when the rupture of the tissue occurred far from the clamps. For this reason, only 12 specimens out of the 19 were considered in the following mechanical analysis.

**Figure 4:**
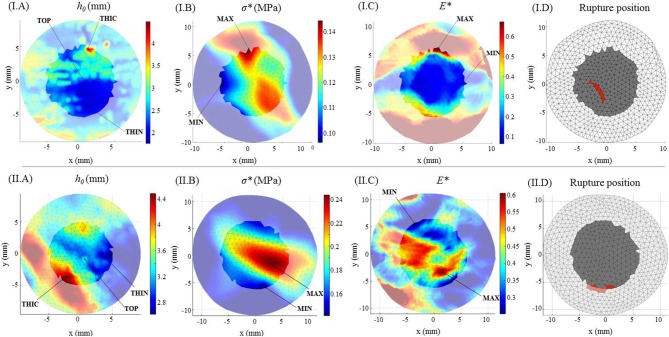
Geometrical mechanical and kinematical fields for specimen no 3 (I) and no 19b (II). Color maps of **(A)** the initial thickness *h*_0_, **(B)** the maximum principal Cauchy membrane stress tensor σ* at 150 mmHg, **(C)** the maximum principal Green Lagrange tensor E* at 150 mmHg. **(D)** Rupture location on the surface (in red) within the ROI mesh (in gray).

**Figure 5:**
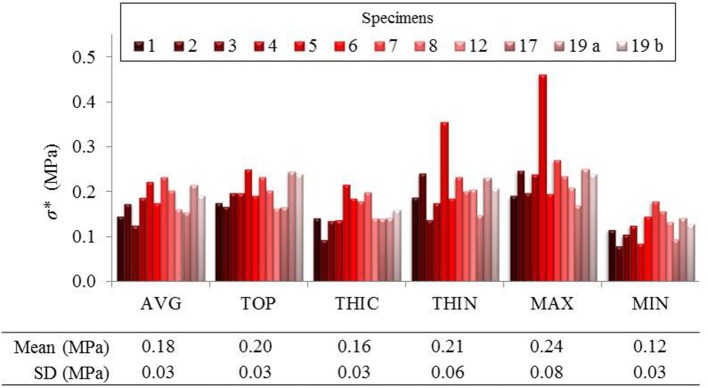
Local maximum principal Cauchy membrane stress σ* reported in average and in 5 different points of the ROI of several specimens.

**Figure 6:**
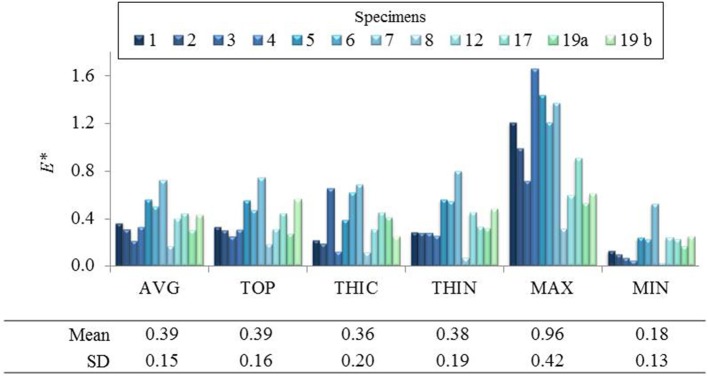
Local maximum principal Green Lagrange strain E* reported in average and in 5 different points of the ROI of several specimens.

The fields of maximum principal value of the Cauchy membrane stress tensor, σ^*^, and of the Green Lagrange tensor, E^*^, were computed and analyzed at the loading state which was equivalent to the *in vivo* pressure of 150 mmHg. The analysis was limited to a region of interest (ROI) of 10 mm diameter from the center of the inflated surface. This permitted to have the complete mechanical field reconstruction for all specimens (complete s-DIC results were not always obtained close to the borders) and to avoid inaccuracy at the borders due to the imposed boundary conditions in the stress reconstruction method (Romo et al., 2014). Figures 4A–C show typical local maps of initial thickness *h*_0_, σ^*^, E^*^ within the ROI of specimen no 3 and 19 b. The high spatial resolution in the measurements resulted in well-defined distributions. It transpires from the maps that the three fields were highly heterogeneous within the ROI, showing unpredictable distributions. For each analyzed specimen, the local values were considered at five different nodal locations of the reconstructed ROI in order to facilitate the analysis: the top (TOP) of the inflated shape, the initial thickest (THIC) and the initial thinnest (THIN) locations, the maximum (MAX), and the minimum (MIN) values locations, as indicated in the examples in Figure 4. Moreover, the all-node average value (AVG) was considered, and displayed in Figures 5, 6. These graphs show that the maximum σ^*^ was in average 2.1 times as high as the minimum σ^*^. In addition, the maximum E^*^ was in average 9.1 times higher than the minimum E^*^. It is important to note that MAX and THIN values, respectively, MIN and THIC, were not equal in most cases.

#### Pointwise Correlation Between Thickness and Mechanical State

It can be observed in Figure 5 that σ^*^ at THIN nodes were higher than the values at THIC nodes in all specimens, in average by +38 ± 43%. Instead, considering E^*^ values at THIN and THIC nodes (Figure 6), no defined relationship was observed between the strain values and the initial thickness: E^*^ was higher in the thinnest than in the thickest nodes for 7 specimens but for 5 specimens the trend was reversed, with an average change of +20 ± 53%. Now considering all nodes in each specimen, it was first found that σ^*^ and thickness were significantly negatively correlated. On the contrary, the relationship between E^*^ and the initial thickness varied among the specimens, as summarized from the correlation coefficients in Table 2. Interestingly, the relationship between E^*^ and thickness was significantly positive in 4 specimens, significantly negative in another 4 and not significant in the rest of the specimens. The table also shows that the two specimens of patient 19 followed opposite trends.

**Table 2:** Correlation (ρ and *p*-value) between the mechanical values σ* and E* and the initial thickness *h*_0_ at all the nodal locations of each specimen.

**Subject/specimen**		**Correlation coefficient ρ (*****p*****-value)**	
		σ*	E*
1		−0.38 (<0.05)	0.03 (0.31)
2		−0.23 (<0.05)	0.04 (0.32)
3		−0.18 (<0.05)	0.15 (<0.05)
4		−0.46 (<0.05)	−0.02 (0.73)
5		−0.51 (<0.05)	−0.56 (<0.05)
6		−0.55 (<0.05)	0.17 (<0.05)
7		−0.42 (<0.05)	−0.53 (<0.05)
8		−0.29 (<0.05)	0.49 (<0.05)
9		−0.77 (<0.05)	−0.82 (<0.05)
17		−0.39 (<0.05)	0.46 (<0.05)
19	a	−0.83 (<0.05)	0.08 (0.08)
	b	−0.87 (<0.05)	−0.21 (<0.05)

*A p-value < 0.05 indicates a significant correlation*.

#### Comparison With the Use of the Specimen's Average Thickness

**Figure 7:**
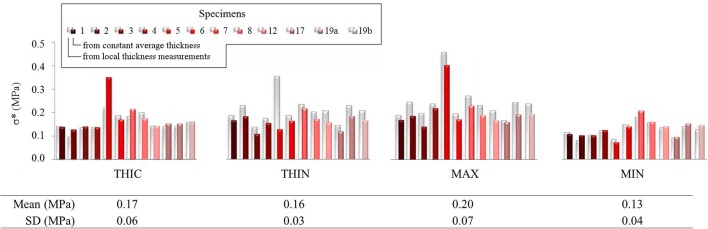
Local maximum principal membrane stress σ* reported in four of the defined characteristic points within the ROI of several specimens. Values calculated using local thickness measurements are represented as gray striped shadows for each histogram column, while those calculated with constant average thickness are in color.

The present analysis was also carried out using a homogeneous thickness value to estimate σ^*^, as performed in all previous works found in the literature. Figure 7 shows the corresponding values of σ^*^ in comparison with the same values calculated with the actual thickness field for node positions THIN, THIC, MAX, and MIN previously defined.

The values indicated important differences of +9.5 ± 23.3%, for THIC (over-estimation), and of −23.0 ± 18.4% for THIN (under-estimation) (average and standard deviation). The range of value defined by $\sigma_{MAX}^{*}-\sigma_{MIN}^{*}$ reduced by −42.9 ± 23.5% when a constant thickness was used, demonstrating the relevance of using the actual thickness field in stress analysis for such tissue.

### Mechanical Analysis at Rupture

All measured rupture pressure values and their equivalent *in vivo* values are listed in Table 3. The measured rupture pressure was 1,014.47 ± 225.02 mmHg, average and standard deviation respectively, for all specimens. When converted to the *in vivo* rupture pressure using Equation (4), the corresponding pressures was 742.73 ± 220.49 mmHg. From this point on, only the *in vivo* pressure was taken into account for the subsequent analysis of the specimens.

**Table 3:** Measured and corresponding *in vivo* rupture pressure values.

**Specimen**		**Rupture pressure (mmHg)**	
		**Measured**	***In vivo***
1		1,335.11	1,081.66
2		969.90	639.13
3		1,263.55	1,078.51
4		1,053.61	750.51
5		1,232.72	869.84
6		1,139.34	813.37
7		1,118.19	842.09
8		1,041.46	781.71
9		890.47	635.68
17		816.29	622.10
19	a	700.41	433.08
	b	613.62	365.80

In addition, the two specimens from the same patient permitted to have comparative results on a same donor. The left specimen of the ATAA ruptured at an *in vivo* pressure of 433.08 mmHg while the right part of the ATAA ruptured at 365.80 mmHg, i.e., a −16% difference.

Very importantly, taking into account the position of the rupture in relation with thickness and physiological mechanical fields, all specimens showed to behave similarly to specimens no 3 and 19 b. It was observed that the location of rupture corresponded to neither a zone of minimal thickness nor a zone where concentration of E^*^ or σ^*^ could be visualized on the color maps (see representative examples in Figure 4).

#### Inter-patient Analysis: Correlation Between Thickness and Rupture Mechanical Data

Considering all specimens together, no significant linear correlation was found between the *in vivo* rupture pressure values and the mean or the minimum thickness of the specimens (*p*-value > 0.45). As it is relevant for clinical implications, we also report that no significant correlation was found between the rupture pressure and any value of σ^*^ under physiological loading, but a significant linear correlation with E^*^ at MAX nodes was found (ρ: 0.56, *p-value*: 0.05).

## Discussion

### Thickness Characterization

A key point of this study was the measurement of local thickness fields instead of a homogeneous value thanks to the development of a novel methodology allowing thickness estimates with a high spatial resolution. This aspect was motivated by the large number of experimental and computational studies having previously shown evidences of regional differences in the elastic and failure mechanical properties of arterial tissue as well as non-uniform distribution of tissue thickness, which were proven to reduce the accuracy of aortic computational models (Choudhury et al., 2009; Iliopoulos et al., 2009b; Kim and Baek, 2011; Shang et al., 2013; Davis et al., 2015; Genovese and Humphrey, 2015; Raaz et al., 2015; Ferraro et al., 2018). The proposed optical technique has the assets of rapidity, specimen preservation, reproducibility with a good accuracy, and a high spatial resolution that common methods of measuring specimen thickness do not allow (Lee and Langdon, 1996; O'Leary et al., 2013). The feasibility of the technique was established here with specimens of unruptured human ATAA wall but this set-up is suitable for other kinds of thin specimens.

The mean thickness which was measured with this setup, of about 3.10 mm, was higher than previous measurements of human unruptured ATAA with similar clinical characteristics. Indeed, multiple measurements conducted with a digital caliper for entire ATAA wall specimens resulted in an average and standard deviation of 1.89 ± 0.28 mm (Romo, 2014), 1.90 ± 0.20 mm (Vorp, 2007) and similar values in other studies (Forsell et al., 2014). The comparison between the measurements carried out with both the method here presented and the digital caliper on a subgroup of four specimens of the study shall clearly define the variable difference. Using the caliper, the average thicknesses were found to be 1.73, 1.84, 2.45, 2.56 mm in specimens 17, 18, 19a, and 19b, respectively, with a reduction of 37, 22, 27, and 23% with respect to the average thickness found with the presented approach. As evidence of this, the accuracy of methods using calipers could be questioned (O'Leary et al., 2013) and the results could be associated with an uncontrolled compression exerted by the user. Using histological slides, Tang et al. (2005) recorded values of 2.60 ± 0.21 mm. Iliopoulos et al. (2009b) carried out both histology and laser micrometry on fresh specimens resulting in 1.63 ± 0.47 mm in fixed specimens but importantly higher (no average reported) in fresh specimens because of fixation and histologic preparation effect which usually is the tissue shrinkage. Smoljkić et al. (2017) reported 1.44 ± 0.28 mm (intima-media thickness without adventitia) through histology and 2.72 ± 0.44 mm (full thickness) via an optical method by placing the specimen between two calibrated metal plates. Other works, relevant for potential clinical use, were based on echocardiographic imaging, but being under-load imaging by nature, a comparison with our results was not possible (Koullias et al., 2005; Nathan et al., 2011).

Unlike earlier techniques, the presented one allows a user-independent thickness measurement without any applied load on the specimen, with no contact on both specimen sides, and hence without compression or alteration in the thickness direction. These assets enabled unprecedented and reliable assessment of thickness variability. Hence, inter-patient variability was found to be higher compared to the standard deviations presented in the previous studies mentioned above. Interestingly, the intra-specimen variability was higher than the inter-specimen one, underlining the heterogeneity of this tissue and providing new evidence to better understand arterial diseases. Though their causes were not of interest in this study, such heterogeneities are known to be induced by several pathologies, e.g., atherosclerosis (intimal hyperplasia, calcification, fat deposition) or cystic medial necrosis (disruption of elastin and smooth muscle cells).

### Mechanical Analysis of Pressurized ATAA Tissue

#### Mechanical State at Physiological Loading and Rupture Pressure

The maximum principal values σ^*^ and E^*^ of the Cauchy membrane stress tensor and of the Green Lagrange tensor, respectively, were analyzed and compared at key locations of the specimens at an *in vivo* pressure of 150 mmHg. The pressure was chosen to represent an elevated systolic aortic pressure, addressing slight (frequent) hypertension. It is in fact known that 60% of people older than 65 years are hypertensive (Zieman et al., 2005) and that hypertension is frequently associated to aneurysm formation (Hameed et al., 2011).

At this *in vivo* pressure, the mean maximum principal stress σ^*^ was 0.18 MPa and the mean maximum principal strain E^*^ was 0.39, in agreement with the wide range of values found in the literature for biaxial tests on same tissues (Okamoto et al., 2002, 2003; Matsumoto et al., 2009; Duprey et al., 2016). Our results thus constitute an additional contribution to the knowledge of the mechanical state with local information.

When increasing the pressure up to the arterial wall rupture, the mean measured rupture pressure for all specimens was 1,014.47 mmHg, which was equivalent to an *in vivo* rupture pressure of 742.73 mmHg. This level of pressure is much higher than the highest physiological aortic pressure reached during the rest cardiac cycle, i.e., of about 120 mmHg (Michal et al., 2015) or slightly higher for hypertensive patients. However, it must be noted that such levels of pressure may be reached in certain physiological circumstances. Early investigations by Kroell et al. (1974) about the mechanical response to impact of cadaver thoraces with initially pressurized aortas revealed intravascular pressures over 2,500 mmHg, with two cases of resulting traumatic rupture of the aorta with burst pressures over 1,500 mmHg. This suggest that considering the intra-aortic pressure is fundamental to determine the risk of rupture when the wall strength is altered, e.g., in case of diseased tissue (Vorp et al., 2003). Unfortunately, there is a lack of data on the actual pressure levels at which ATAA rupture *in vivo*. Therefore, bulge inflation is a good compromise to test excised flat strips up to rupture. This test was first performed by Mohan and Melvin (1983) who studied human descending aortic tissue but without reporting values of rupture pressure of their tests. A similar test was performed on porcine aortas by Marra et al. (2006) who reported a rupture pressure of 1,500 mmHg in one representative porcine specimen. However, these data may not be compared to human ATAA. Tests of bulge inflation using specimens of human non-aneurysmal ATAAs with intima outwards were later carried out by Kim et al. (2012) followed by Romo (2014). The latter indicated for 23 specimens of 11 patients a mean measured rupture pressure of 731 ± 309 mmHg. Their values were lower than the presented ones and this may be partly due to their inflation technique with the intima layer outwards.

#### Pointwise Mechanical Analysis Including Accurate Thickness

The most relevant and original contribution of this work lies in the combined analysis of pointwise mechanical in-plane quantities (stress and strain) and thickness, which both exhibited strong, and uncorrelated spatial heterogeneities. The range of maximum principal stress σ^*^ at a physiological *in vivo* pressure of 150 mmHg was between 0.12 and 0.25 MPa and that of maximum principal strain E^*^ ranged between 0.12 and 0.95, highlighting a marked local heterogeneity of the mechanical properties. Previous studies already underlined this point with similar experiments on ATAAs (Romo et al., 2014; Davis et al., 2015, 2016; Trabelsi et al., 2015; Duprey et al., 2016) or other biaxial tests on aortic tissue (Bersi et al., 2016; Peña et al., 2018). However, the major limitation of the aforementioned studies was to assume a homogeneous wall thickness, which could alter the calculation of stresses as clearly emphasized from Figure 7. Indeed, carrying out the same analysis using a homogeneous (average) thickness instead of the local thickness measurements, we demonstrated that the maximum principal stress range underwent a visible decrease. In particular, the thinnest nodes experienced the largest stress underestimation. In the eventuality that ATAAs failure is associated to a local thickness particularity, or is stress-driven, the contribution of thickness information at high spatial resolution could be a major step forward.

Furthermore, we found various trends when analyzing, specimen by specimen, all pointwise data together, the mechanical quantities with respect to thickness. While the maximum principal stress was negatively correlated to the thickness as expected, the maximum principal strain showed either positive or negative, most often significant, correlation. This inconsistent strain response was also observed when comparing two specimens extracted from the same aneurysm. These results imply that the local mechanical variations are most likely due to the intrinsic nature and organization of the microstructural components within the arterial wall of each specimen.

With respect to rupture pressure, no significant correlation was found between the equivalent *in vivo* rupture pressure and the specimen mean or minimum thicknesses, or maximum stress values. As a consequence, these observations suggest that the arterial wall strength, elongation at rupture, and hence microstructure may differ a lot from a patient to another, because neither the thickness alone, the stress or the strain could be used as relevant markers of the rupture location. Consistently, in uniaxial tensile tests, some authors did not find significant correlation between ultimate wall strength and mean thickness of the strips (Ferrara et al., 2018); on the contrary, other authors reported a trend toward a significant negative correlation (Iliopoulos et al., 2009a; Khanafer et al., 2011;Forsell et al., 2014).

These results have direct implications for the biomechanical analysis of aneurysms. Thickness heterogeneity of arterial tissues was a topical discussion (Davis et al., 2016; Farotto et al., 2018). With this study, accurate local thickness measurement was a benefit in providing detailed mechanical analyses of pressurized ATAA tissue. Although a better knowledge on the microscopic origin of thickness variations is needed, this work may already have important practical implications concerning the clinical management of patients at risk of rupture. Indeed, computational diagnosis methods (Gasser, 2016) are emerging which focus on computing case-specific wall stress. Our results demonstrate that precisely knowing the local thickness would not be decisive in mechanically assessing the severity of a single aneurysmal case with such methods. Instead the accurate local thickness measurements performed in this study raise the need for a better knowledge of the origin of strength variations, suggesting that intravascular microstructural exams may prove relevant in future clinical practice.

This study has some limitations and the following improvements could be addressed to confirm, reinforce and extend our findings. First, human ATAA tissues are complex to analyze statistically, as age and many potential comorbidities can be confounding factors. A higher number of specimens and a more normal distribution of the population clinical data are needed to provide additional robustness to the presented statistical results. On a similar aspect, the study should be extended to include non-aneurysmal specimens, in order to better analyze the effects of the pathology over the aging effects. As a promising perspective, a characterization of the principal microstructural constituents of the ATAA wall along with their dynamic evolution in the process of inflation and rupture of the tissue will shortly contribute in shedding light on the local mechanical variability issue.

## Conclusions

Understanding the interplay between the heterogeneous thickness and the microstructure of ATAAs and their mechanical behavior up to failure is a topical subject with practical implications concerning the computational mechanics and its applications in clinics. The most original and remarkable outcome of this study is the absence of a clear link between the local wall thickness and the stress-strain state in ATAAs at physiological and rupture pressures. The analyses we carried out at both the subject and the population levels suggest that the knowledge of the wall thickness—even if measured at a high spatial resolution—is insufficient to properly describe the physiological and extreme mechanical states. The need for investigating the microstructure of the aneurysmal arterial wall in the dynamic mechanical state is thus recognized as a primary necessity and it will the objective of related future studies.

## Data Availability

The raw data supporting the conclusions of this manuscript will be made available by the authors, without undue reservation, to any qualified researcher. Requests to access the datasets should be directed to BP, badel@emse.fr

## Ethics Statement

This study was carried out in accordance with the French regulations and approved by the ministry of research, with written informed consent from all subjects. All subjects gave written informed consent in accordance with the Declaration of Helsinki. The protocol was approved by the ethics committee France Sud-Est 1.

## Author Contributions

All authors made substantial contributions to the study and gave final approval for submission. CC, JM, NC, and PB contributed to the design of the thickness measurement device. SC contributed to tissue dissection and clinical data collection. CC, JM, LO, and PB contributed to the conception and design of the study, analysis, interpretation of the data and critical revision.

### Conflict of Interest Statement

The authors declare that the research was conducted in the absence of any commercial or financial relationships that could be construed as a potential conflict of interest.
